# High-Throughput Assay Development for Cystine-Glutamate Antiporter (x_c_^-^) Highlights Faster Cystine Uptake than Glutamate Release in Glioma Cells

**DOI:** 10.1371/journal.pone.0127785

**Published:** 2015-08-07

**Authors:** Ajit G. Thomas, Rita Sattler, Karen Tendyke, Kara A. Loiacono, Hans Hansen, Vishal Sahni, Yutaka Hashizume, Camilo Rojas, Barbara S. Slusher

**Affiliations:** 1 Brain Science Institute, Johns Hopkins University School of Medicine, Baltimore, MD, 21205, United States of America; 2 Department of Neurology, Johns Hopkins University School of Medicine, Baltimore, MD, 21205, United States of America; 3 Next Generation Systems CFU, Eisai Inc., Andover, MA, 01810, United States of America; 4 Neuroscience and General Medicine PCU, Eisai Inc., Andover, MA, 01810, United States of America; 5 Department of Comparative Medicine and Molecular Pathobiology, Johns Hopkins University School of Medicine, Baltimore, MD, 21205, United States of America; 6 Department of Psychiatry, Johns Hopkins University School of Medicine, Baltimore, MD, 21205, United States of America; 7 Department of Neuroscience, Johns Hopkins University School of Medicine, Baltimore, MD, 21205, United States of America; University of Cambridge, UNITED KINGDOM

## Abstract

The cystine-glutamate antiporter (system x_c_^-^) is a Na^+^-independent amino acid transporter that exchanges extracellular cystine for intracellular glutamate. It is thought to play a critical role in cellular redox processes through regulation of intracellular glutathione synthesis via cystine uptake. In gliomas, system x_c_^-^ expression is universally up-regulated while that of glutamate transporters down-regulated, leading to a progressive accumulation of extracellular glutamate and excitotoxic cell death of the surrounding non-tumorous tissue. Additionally, up-regulation of system x_c_^-^ in activated microglia has been implicated in the pathogenesis of several neurodegenerative disorders mediated by excess glutamate. Consequently, system x_c_^-^ is a new drug target for brain cancer and neuroinflammatory diseases associated with excess extracellular glutamate. Unfortunately no potent and selective small molecule system x_c_^-^ inhibitors exist and to our knowledge, no high throughput screening (HTS) assay has been developed to identify new scaffolds for inhibitor design. To develop such an assay, various neuronal and non-neuronal human cells were evaluated as sources of system x_c_^-^. Human glioma cells were chosen based on their high system x_c_^-^ activity. Using these cells, [^14^C]-cystine uptake and cystine-induced glutamate release assays were characterized and optimized with respect to cystine and protein concentrations and time of incubation. A pilot screen of the LOPAC/NINDS libraries using glutamate release demonstrated that the logistics of the assay were in place but unfortunately, did not yield meaningful pharmacophores. A larger, HTS campaign using the 384-well cystine-induced glutamate release as primary assay and the 96-well ^14^C-cystine uptake as confirmatory assay is currently underway. Unexpectedly, we observed that the rate of cystine uptake was significantly faster than the rate of glutamate release in human glioma cells. This was in contrast to the same rates of cystine uptake and glutamate release previously reported in normal human fibroblast cells.

## Introduction

The cystine-glutamate antiporter (system x_c_^-^) is a Na^+^-independent amino acid transporter that exchanges extracellular cystine for intracellular glutamate [1]. Since cystine is a precursor for glutathione (GSH), system x_c_^-^ is believed to play a critical role in intracellular GSH synthesis and subsequent cellular redox regulation [2]. Additionally, the release of glutamate into the extracellular space, via the antiporter, makes system x_c_^-^ a key determinant of extracellular glutamate concentrations.

System x_c_^-^ is thought to play a significant role in the pathogenesis of cancer and neurodegenerative diseases. In gliomas, system x_c_^-^ expression is universally up-regulated and glutamate transporters are down-regulated, leading to a progressive accumulation of extracellular glutamate, thereby causing peritumoral seizures and excitotoxic cell death to surrounding neurons [3]. This provides a significant growth advantage to the tumor by offering space for expansion [4–7]. Additionally, glioma cells have a unique requirement for extracellular cystine, as they tend to lack the ability to synthesize cysteine [8]. This renders extracellular cystine uptake critical for glutathione synthesis and thus, tumor survival and growth [2, 9–11]. In fact, inhibitors of system x_c_^-^ have been shown to significantly reduce brain tumor growth, block abnormal EEG and seizure activity, attenuate perifocal edema and increase survival in animal models [9, 12].

Glutamate release has also been shown to be mediated via system x_c_^-^ in activated microglia [13, 14]. Given the antiporter’s potential involvement in glutamate excitotoxicity, up-regulation of system x_c_^-^ in activated microglia has also been implicated in the pathogenesis of many neurodegenerative disorders [15], including Alzheimer's disease [16], Parkinson's disease [17], HIV-associated neurocognitive disorders [18], multiple sclerosis [19] and epilepsy [20].

Considering the potential role of system x_c_^-^ in cancer and neurodegenerative diseases, as well as the validation of the target via both genetic (with siRNA) and pharmacological (with prototype small molecules) inhibition of the antiporter in pre-clinical models [9, 12], it is of interest to develop system x_c_^-^ inhibitors that could be evaluated in the clinic. While a number of small molecule system x_c_^-^ inhibitors has been described, none have shown potency and selectivity for the target [21–25]. Several prototype antiporter inhibitors are glutamate mimics, such as (*S*)-4-carboxyphenylglycine ((*S*)-4CPG), but they also have activity at various glutamate receptors and hence lack specificity [23, 24]. Sulfasalazine (SAS), a FDA approved drug for the treatment of Crohn’s disease, is also an inhibitor of system x_c_^-^ [26]. However, SAS lacks selectivity as it possesses both anti-inflammatory and antibacterial activity and inhibits NF-κB [27]. SAS is also extensively metabolized *in vivo* by intestinal bacteria to 5-aminosalicylic acid and sulfapyridine, both metabolites being inactive against system x_c_^-^ [26]. Therefore, to fully exploit the therapeutic potential of this target, it is critical to identify new structural entities that potently and selectively inhibit system x_c_^-^. To our knowledge, no high throughput screening (HTS) assay has been developed for this target. Various assays have been used to identify system x_c_^-^ activity including cystine [1, 3, 28] or glutamate uptake [7, 24] and cystine-induced glutamate release [7, 24]. Here, we describe the characterization of both a cystine uptake assay and a cystine-induced glutamate release assay for implementation in HTS using human astrocytoma cells. We also show that the rate of uptake of cystine mediated by system x_c_^-^ is approximately 10 to 14-fold higher than the rate of glutamate release in human glioma cells, which is in contrast to previous studies performed in normal human fibroblast [29].

## Materials and Methods

### Reagents

Unless otherwise stated, all chemicals and glutamate dehydrogenase (GDH) were obtained from Sigma-Aldrich (Sigma, St. Louis, MO).

### Cell lines

Daudi (lymphoblast), CCF-STTG-1 (astrocytoma), H4 (neuroglioma), IMR-90 (fibroblast), THP-1 (monocytes), U-87 MG (glioblastoma), obtained from American Type Culture Collection (ATCC, Manassas, VA) and P-493 B (lymphoma) cells, obtained from Dr. Chi Dang (Abramson Cancer Center, University of Pennsylvania, Philadelphia, PA; *Nature* **458**, 762–765, 2009), were examined for system x_c_^-^ activity. The Daudi, P-493 B, CCF-STTG-1 and THP-1 cells were grown in RPMI-1640 media (ATCC: 30–2001) containing 10% fetal bovine serum (FBS, ATCC: 30–2020) and 1% Antibiotic-Antimycotic (Ab-Am; Life Technologies, Grand Island, NY: 15240062). Growth media for THP-1 cells also included 0.05 mM β-mercaptoethanol. U-87 MG cells were grown in Eagle’s MEM (EMEM, ATCC: 30–2003) containing 20% FBS and 1% Ab-Am, while IMR-90 cells were grown in EMEM with 10% FBS and 1% Ab-Am. Finally, the H4 cells were grown in Ham’s F-12 Nutrient Mix, GlutaMax supplement (Life Technologies: 31765035) with 10% FBS and 1% Ab-Am.

### Quantitative Reverse Transcription-PCR

Total RNA was isolated from the various cells using the RNEasy Mini kit (Qiagen, Valencia, CA) and converted to cDNA using the High Capacity cDNA Reverse Transcription kit (Applied Biosystems, Foster city, CA). Quantitative PCR was completed using TaqMan pre-made gene-specific probes designed against the light chain subunit of system x_c_^-^, specifically xCT or SLC7A11 (the catalytic unit of system x_c_^-^, conjugated to FAM reporter dye; Applied Biosystems: 4331182) and against the endogenous control, 18S ribosomal RNA (conjugated to VIC reporter dye; Applied Biosystems: 4319413E). The relative abundance of xCT in the various cell lines was determined using the 2^-ΔΔCT^ method [30, 31] and presented as a percent of the levels in the human astrocytoma (CCF-STTG-1) line.

### [^14^C]-Cystine uptake assay

Cystine uptake procedures were adapted from previously published reports [3]. Early characterization work was carried out in a 12-well format and the assay later adapted and characterized in the 96-well format. Briefly, CCF-STTG-1 cells were seeded at 0.04 x 10^6^ cells per well in 0.2 ml growth media and grown to confluence. On the day of the experiment, cells were washed three times (100 μl/well) with pre-warmed (37°C) chloride-dependent, sodium-independent, uptake buffer (UB, contents in mM: choline chloride 137.5, KCl 5.36, KH_2_PO_4_ 0.77, MgSO_4_ 0.71, CaCl_2_ 1.1, Glucose 10 and HEPES 10, pH 7.4) and the kinetics of uptake initiated upon the addition of L-[3,3’-^14^C]-cystine (PerkinElmer, Waltham, MA: NEC845050UC) in buffer, at 37°C, containing 0–400 μM cystine (5.631 μCi/μmol). After 15 min, uptake was terminated by three washes (115 μl/well) with ice-cold UB. Cells were then lysed with 0.1 N NaOH (100 μl/well) and the radioactivity in the cells measured using scintillant-coated 96-well plates (PerkinElmer: 6005630) and normalized to protein contents. The assay was further characterized for the dependence of ^14^C-cystine uptake on protein content (mg/ml) and the time and temperature of incubation. To determine IC_50_ values, CCF-STTG-1 cells were similarly seeded and grown to confluence. On day 3, transport was initiated upon the addition of pre-warmed (37°C) [^14^C]-cystine 80 μM (5.631 μCi/μmol) in Earle’s Balanced Salt Solution (EBSS, Sigma: E3024, contents in mM: NaCl 116.4, NaHCO_3_ 26.2, NaH_2_PO_4_ 1.02, KCl 5.36, MgSO_4_ 0.81, CaCl_2_ 1.8 and Glucose 5.56, pH 7.4) in the presence and absence of inhibitors. Uptake was terminated after 15 min with washes of ice-cold EBSS, the cells lysed with 0.1 N NaOH and radioactivity in cells measured as previously mentioned. The data were then normalized to the ‘totals’ (uptake at 37°C in the absence of inhibitor) and ‘blanks’ (either uptake at 37°C in the presence of 1 mM SAS (96-well format) or uptake at 0°C in the absence of inhibitor (12-well format)) and presented as percent inhibition. Identical protocols were followed for IC_50_ determinations using UB or phosphate buffered saline (PBS; contents in mM: S1 Table) with CCF-STTG-1, neuroglioma (H4) or fibroblast (IMR-90) cells. When using human glioblastoma cells (U-87 MG) as the source of the antiporter, cells were seeded at 0.06 x 10^6^ cells per 96-well in 0.3 ml growth media. On day 2, cystine uptake was conducted in the presence and absence of inhibitors as described previously. At the end of the experiment, cells were lysed and the radioactivity in cells measured using scintillant-coated 96-well plates and normalized to protein contents.

### Cystine-induced glutamate release assay

Early characterization of the cystine-induced glutamate release assay was carried out in the 96-well format and later adapted to the 384-well format. Briefly, CCF-STTG-1 cells were seeded at 0.04 x 10^6^ cells per 96-well and grown to confluence. On Day 3, cells were washed with pre-warmed (37°C) EBSS and transport initiated upon the addition of 0–400 μM cystine in buffer at 37°C. Cells were maintained for 2h at 37°C in an incubator with 5% CO_2_. At the end of the incubation period, cystine-induced glutamate release was measured directly. The assay was further characterized for the dependence of cystine-induced glutamate release on protein content (mg/ml) and the time of incubation. For IC_50_ determinations, cells were also seeded at 0.04 x 10^6^ cells per 96-well (in 0.2 ml growth media) and on Day 3, cells were washed with pre-warmed (37°C) EBSS and transport initiated upon the addition of 80 μM cystine in the presence and absence of inhibitors. Cells were maintained at 37°C in an incubator with 5% CO_2_ and the glutamate released over a 2h period measured. Identical protocols were followed for IC_50_ determinations using UB or PBS with CCF-STTG-1, neuroglioma (H4) or fibroblast (IMR-90) cells. U-87 MG cells were seeded at 0.06 x 10^6^ cells per 96-well (in 0.3 ml growth media) and tested on Day2. When running the assay in the 384-well format, CCF-STTG-1 cells were seeded at 0.01 x 10^6^ cells per well (in 0.025 ml growth media) and grown to confluence. Upon reaching confluence (Day 3), cells were washed (4 x 50 μl/wash) with room temperature EBSS using either Beckman Coulter’s (Biomek NX^P^) or Tecan’s (Freedom EVO150) liquid handling system and transport initiated upon the addition of 80 μM cystine (20 μl). Cells were maintained for 3h at 37°C in an incubator with 5% CO_2_. At the end of the incubation period, cystine-induced glutamate release was measured directly.

### Glutamate measurement

Cystine-induced glutamate released from cells was measured by coupling the effluxed glutamate to either glutamate dehydrogenase (GDH) [32] or glutamate oxidase (GO) [33]. When using GDH (5 U/mL, Sigma: G7882), the reaction was carried out in the presence of nicotinamide adenine dinucleotide phosphate (NADP^+^, 500 μM) to catalyze the conversion of glutamate to α-ketoglutarate, ammonia and NADPH (ex 340, em 460). When using GO (0.04 U/mL, US Biological, Swampscott, MA: G4001-01), the reaction was carried out in the presence of molecular oxygen and water, to catalyze the conversion of glutamate to α-ketoglutarate, ammonia and hydrogen peroxide. In turn, hydrogen peroxide was detected with Amplex UltraRed (50 μM, Life Technologies: A36006), in a reaction catalyzed by horse radish peroxidase (HRP; 0.125 U/mL, Worthington Biochemical, Lakewood, NJ: LS006476), to produce highly fluorescent resorufin (ex 530, em 590). Both GDH and GO assays were conducted in Tris buffer (100 mM, pH 7.4) and rate of change of fluorescence (RFU/s) monitored using Molecular Device’s Spectramax Gemini XPS fluorimeter.

### Libraries for screening

Two libraries of compounds were used initially to confirm that the logistics of the cystine-induced glutamate release were in place. (i) The Library of Pharmacologically Active Compounds, LOPAC^1280^, (Sigma: LO4100-1EA) comprising 1,280 bioactive small molecules and approved drugs from all major drug classes and (ii) the National Institute of Neurological Disorders and Stroke (NINDS) Custom Collection (MicroSource Discovery Systems, CT) comprising 1,040 compounds, mostly FDA-approved and marketed drugs. Test compounds were made up in 100% DMSO and diluted 500-fold to minimize DMSO exposure on cells to 0.2%. Also, both ‘blanks’ (buffer) and ‘totals’ (buffer containing cystine 80 μM) contained 0.2% DMSO to compensate for DMSO-solubilized compounds. Data were normalized to the totals and blanks and IC_50_ values determined as a function of the normalized values. Finally, since cystine-induced glutamate release assay uses GO and HRP for glutamate detection, compounds being evaluated for inhibition of system x_c_^-^ activity could give false positive results as a consequence of inhibiting one of the two coupling enzymes. In order to rule out inhibition of the coupling enzymes, a counter screen was carried out in the absence of cells (the source of system x_c_^-^) but in the presence of both cystine and glutamate.

### Data analysis

The *K*_*m*_ values were determined using GraphPad Prism employing least-squares fit of the Michaelis-Menten equation: *v* = *V*_*max*_[S]/(*K*_*m*_ + [S]). Lineweaver-Burk plots were used to illustrate results from the least-squares fit. The half maximal inhibitory constant (IC_50_) was also determined using GraphPad Prism. Data were normalized to the ‘totals’ (maximum transport or glutamate release) and ‘blanks’ (minimum transport or glutamate release) and IC_50_ values calculated as a function of the normalized values.

## Results

### System x_c_^-^ expression and activity is highest in glioma cells

Assessment of the mRNA expression of the catalytic subunit of system x_c_^-^ (xCT/*SLC7A11*) with the cystine-induced glutamate release activity (see below; expressed here as RFU/s per mg protein) in various non-neuronal, non-adherent human cells (Daudi, P-493 B, THP-1), non-neuronal, adherent human cells (IMR-90) and neuronal, adherent human cells (CCF-STTG-1, H4, U-87 MG) showed a correlation coefficient of 0.96 between *SLC7A11* mRNA levels and activity (Table 1). The highest mRNA and functional transport activity levels were found in the human, neuronal lines, specifically in the neuroglioma (H4) and the grade IV astrocytoma (CCF-STTG-1) cells. Further characterization studies were carried out using these adherent astrocytoma (CCF-STTG-1) cells.

**Table 1:** xCT/*SLC7A11* mRNA and cystine-induced glutamate release activity in various cell lines.

Cell line	xCT/*SLC7A11* mRNA (relative to CCF-STTG-1 cells)	System x_c_^-^ activity (RFU.s^-1^.mg^-1^)
**H4** (human neuroglioma)	172.55	13.76
**CCF-STTG-1** (human astrocytoma)	100.00	6.20
**IMR-90** (human fibroblast)	17.90	3.40
**THP-1** (human monocytes)	8.85	1.31
**DAUDI** (human lymphoblast)	4.31	3.49
**P-493 B** (human lymphoma)	3.82	1.34
**U-87 MG** (human glioblastoma)	1.23	0.31

### [^14^C]-Cystine uptake is dependent on cystine and protein concentrations and time of incubation

**Fig 1:**
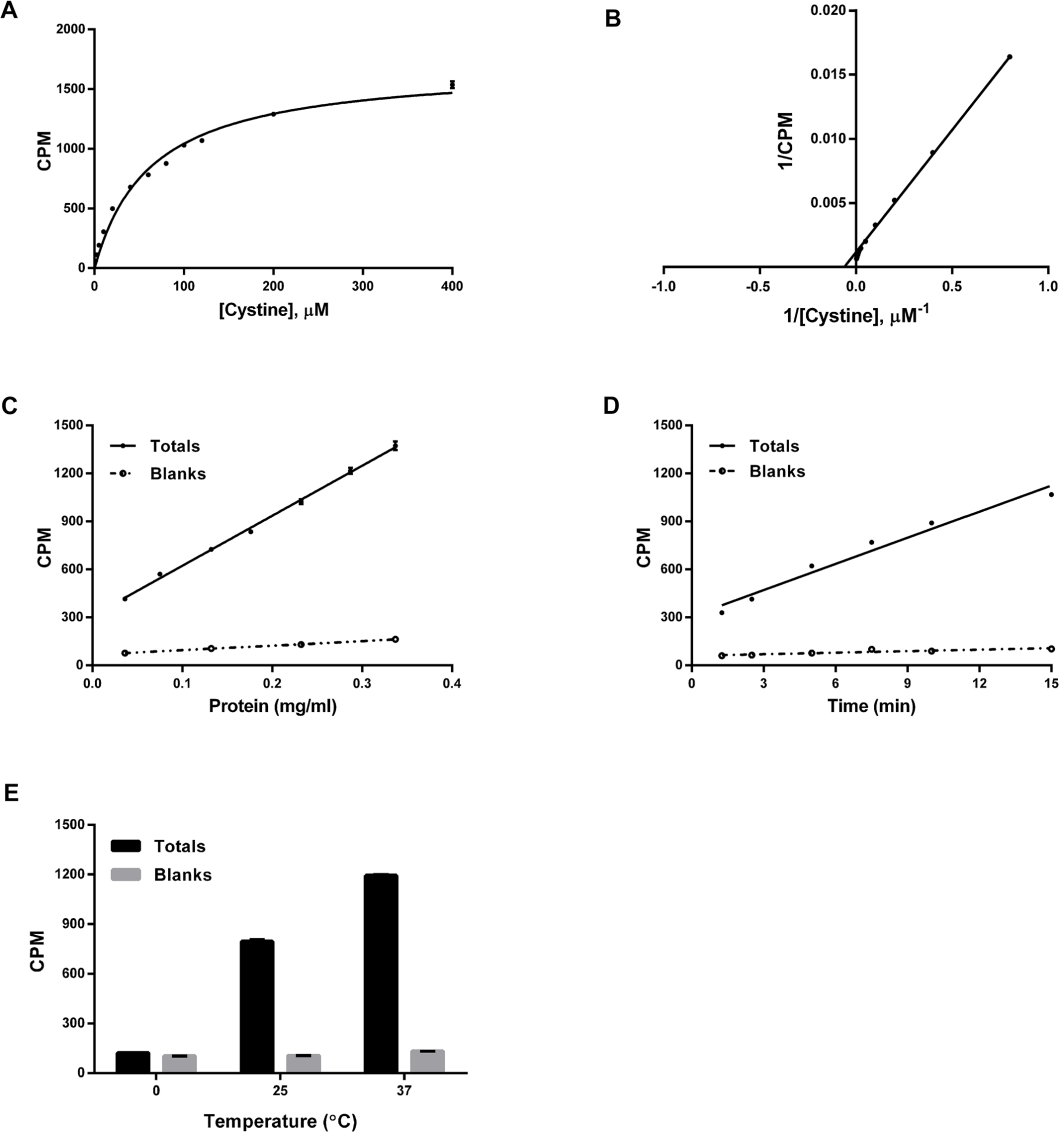
Dependence of [^14^C]-cystine uptake in CCF-STTG-1 cells on A. cystine concentration, B. cystine concentration represented via Lineweaver-Burk transformation, C. protein concentration (mg/ml), D. time and E. temperature of incubation. Unless otherwise specified, CCF-STTG-1 cells were seeded at 0.04 x 10^6^ cells per 96-well, grown to confluence (Day 3), washed with pre-warmed chloride-dependent, sodium-independent, uptake buffer and transport initiated upon the addition of L-[3,3’-^14^C]-cystine at a specific activity of 5.631 μCi/μmol. Substrate dependence experiments were carried out with 0–400 μM cystine while protein-, time- and temperature-dependence experiments were carried out with 80 μM cystine. Except for the time-dependence experiments, uptake was terminated after 15 min with washes of ice-cold uptake buffer. Subsequently, cells were lysed with 0.1 N NaOH and the radioactivity in the cells measured using a liquid scintillation counter and normalized to the protein contents. Data are an average of 2 or more independent experiments with 16–24 determinations per experiment.

Substrate dependence using CCF-STTG-1 cells was initiated upon the addition of 0–400 μM L-[^14^C]-cystine. The uptake of cystine through system x_c_^-^ was linear at lower cystine concentrations and leveled off at the higher cystine concentrations, an indicator of transporter saturation (Fig 1A). Non-linear analysis for the apparent *K*_*m*_ yielded a concentration of 63 ± 4 μM, illustrated by the Lineweaver-Burk transformation (Fig 1B). The uptake of cystine was linear with respect to protein (mg/ml, Fig 1C) and time of incubation (Fig 1D). Additionally, cystine uptake was sensitive to the temperature of incubation, with no transport at 0°C and with increased uptake occurring at higher temperatures (Fig 1E).

### Cystine-induced glutamate release is dependent on cystine and protein concentrations and time of incubation

#### Glutamate measurement

Glutamate efflux from CCF-STTG-1 cells as measured by GDH (S1A Fig) and GO (S1B Fig) resulted in similar IC_50_ values but with significantly improved specific rates (difference between ‘blanks’ (buffer) and ‘totals’ (buffer with cystine)) and *Z’* values when using GO based assay (S1B Fig). Subsequent glutamate release assays were conducted with glutamate metabolism coupled to GO.

#### Assay characterization

**Fig 2:**
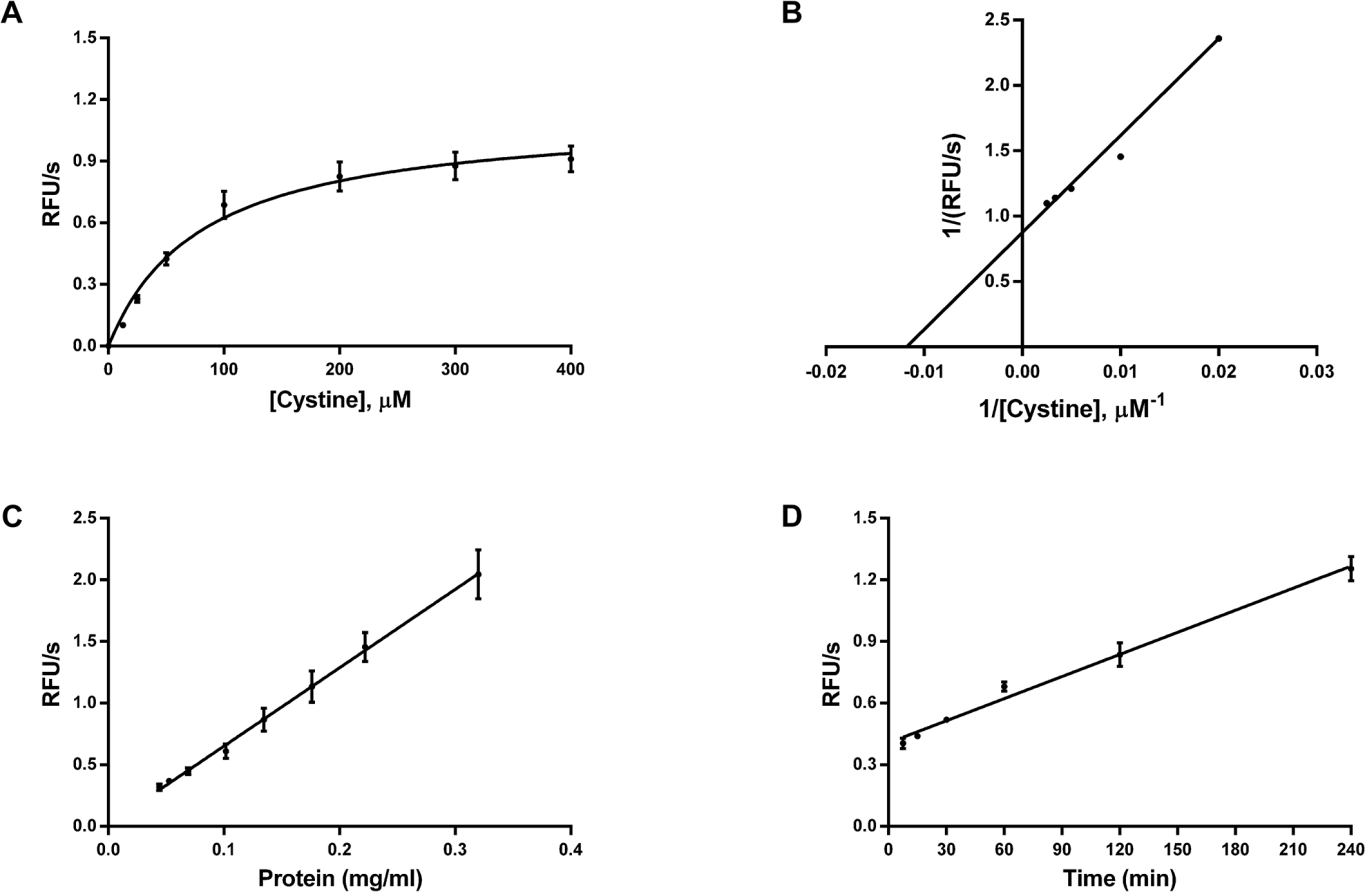
Dependence of cystine-induced glutamate release in CCF-STTG-1 cells at 37°C on A. cystine concentration, B. cystine concentration represented via Lineweaver-Burk transformation C. protein concentration (mg/ml) and D. time of incubation. Unless otherwise specified, CCF-STTG-1 cells were seeded at 0.04 x 10^6^ cells per 96-well, grown to confluence (Day 3), washed with pre-warmed EBSS and transport initiated upon the addition of cystine. Substrate dependence experiments were carried out with 0–400 μM cystine while protein- and time-dependence experiments were carried out with 80 μM cystine. Cells were maintained for 2h at 37°C in an incubator with 5% CO_2_. At the end of the incubation period, glutamate release was measured directly using glutamate oxidase (0.04 U/mL), HRP (0.125 U/mL) and Amplex UltraRed (50 μM), in Tris buffer (100 mM, pH 7.4), at ex 530, em 590. Data are an average of 3 independent experiments with 16–24 determinations per experiment.

**Fig 3:**
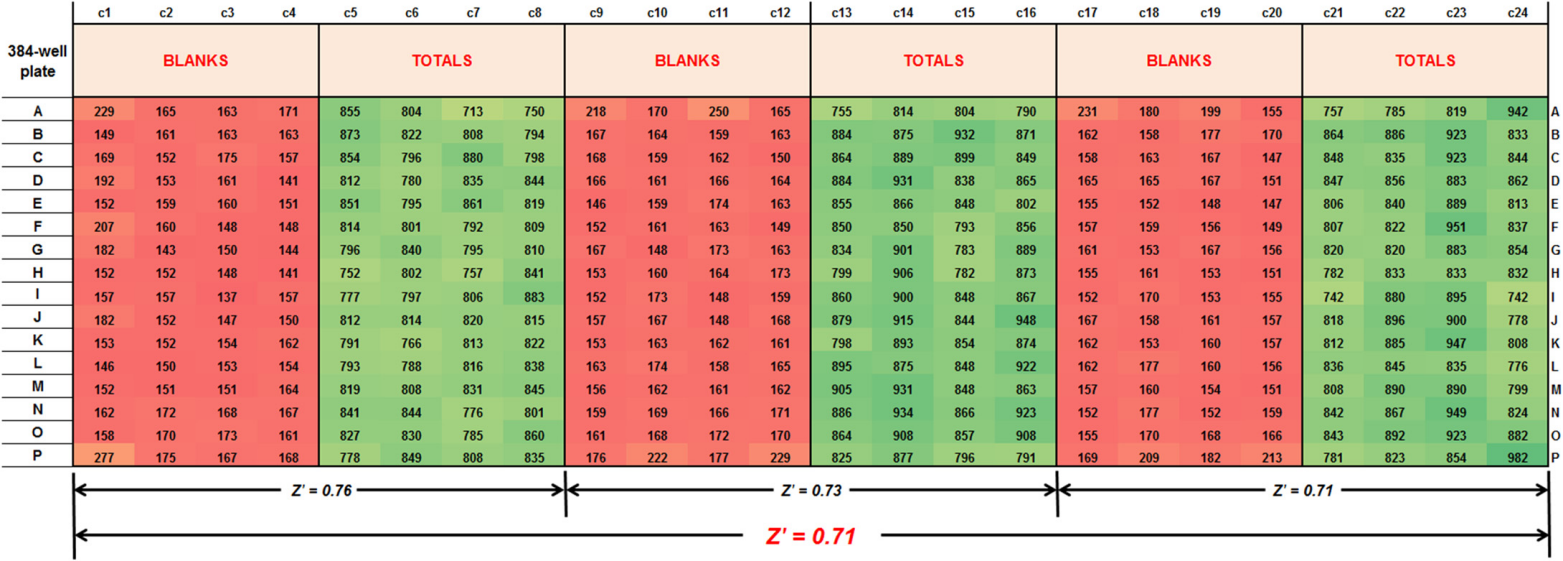
Heat map of 384-well cystine-induced glutamate release assay variability post-optimization. Uniformity in color, for both blanks and totals, illustrates the *Z’* values. CCF-STTG-1 cells were seeded at 0.01 x 10^6^ cells per 384-well in 0.025 ml growth media and grown to confluence. On Day 3, cells were washed (4 x 50 μl/wash) with EBSS (at RT) using either Beckman Coulter’s (Biomek NX^P^) or Tecan’s (Freedom EVO150) liquid handling systems and transport initiated upon the addition of 80 μM cystine (20 μl). Cystine-induced glutamate release after 3h at 37°C was measured upon the addition of 25 μl of glutamate oxidase (0.04 U/mL), HRP (0.125 U/mL) and Amplex UltraRed (50 μM) in Tris buffer (100 mM, pH 7.4) and fluorescence followed at ex 530, em 590.

Substrate dependence in CCF-STTG-1 cells was initiated upon the addition of 0–400 μM cystine. Cystine-induced glutamate release through system x_c_^-^ exhibited Michaelis-Menten behavior: glutamate release was linear at lower cystine concentrations and leveled off at the higher cystine concentrations (Fig 2A) with an apparent *K*_*m*_ of 84 ± 12 μM and illustrated by the Lineweaver-Burk plot (Fig 2B). Cystine-induced glutamate release was linear up to a plating density of 60,000 cells/well (0.320 mg/ml; Fig 2C) and to an incubation time of 240 min (Fig 2D). Finally, optimization of glutamate-release assays in the 384-well format resulted in *Z’* values ranging from 0.5–0.7 across both different sections of the plate and the entire plate (Fig 3).

### Cystine-induced glutamate release assay was used to screen the LOPAC and NINDS chemical libraries

**Fig 4:**
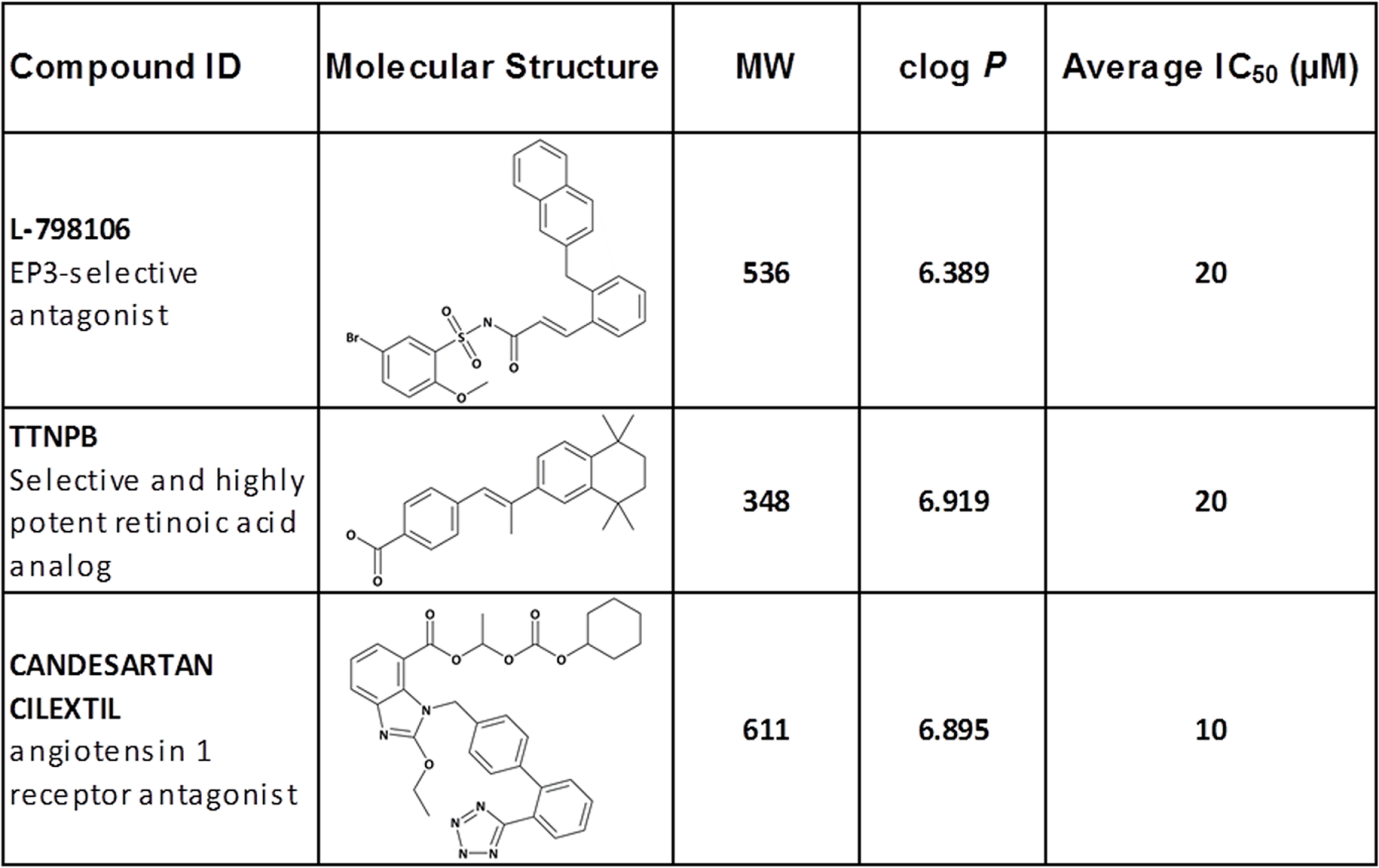
Initial screening results of the compounds in the LOPAC and NINDS chemical libraries using the cystine-induced glutamate release assay. CCF-STTG-1 cells were seeded and grown to confluence. On Day 3, cells were washed with pre-warmed EBSS and transport initiated upon the addition of cystine (80 μM). Cystine-induced glutamate released over 2h at 37°C, in the presence and absence of inhibitors, was measured directly using glutamate oxidase, horse radish peroxidase and Amplex UltraRed and the rate of change of fluorescence monitored at ex 530, em 590. Results were normalized to totals and blanks and the IC_50_ values determined as a function of the normalized values.

An initial activity analysis using ‘totals’ (buffer containing cystine 80 μM), with and without 0.2% DMSO, gave similar rates of change of fluorescence (S2 Table). Consequently, test compounds were made up at 500X the final concentration in 100% DMSO to limit DMSO exposure on cells to 0.2%. Compounds in the LOPAC and NINDS libraries were evaluated for system x_c_^-^ activity at an initial concentration of 20 μM using the cystine-induced glutamate release assay. Out of 2320 compounds, 35 inhibited system x_c_^-^ activity by 50% or higher. After excluding compounds that were auto fluorescent and fluorescence quenchers, a counter screen was carried out to detect inhibitors of either glutamate oxidase or HRP. The hits are shown in Fig 4.

### IC_50_ values in cystine-induced glutamate release assays are lower than those in cystine uptake assays within each cell line

**Fig 5:**
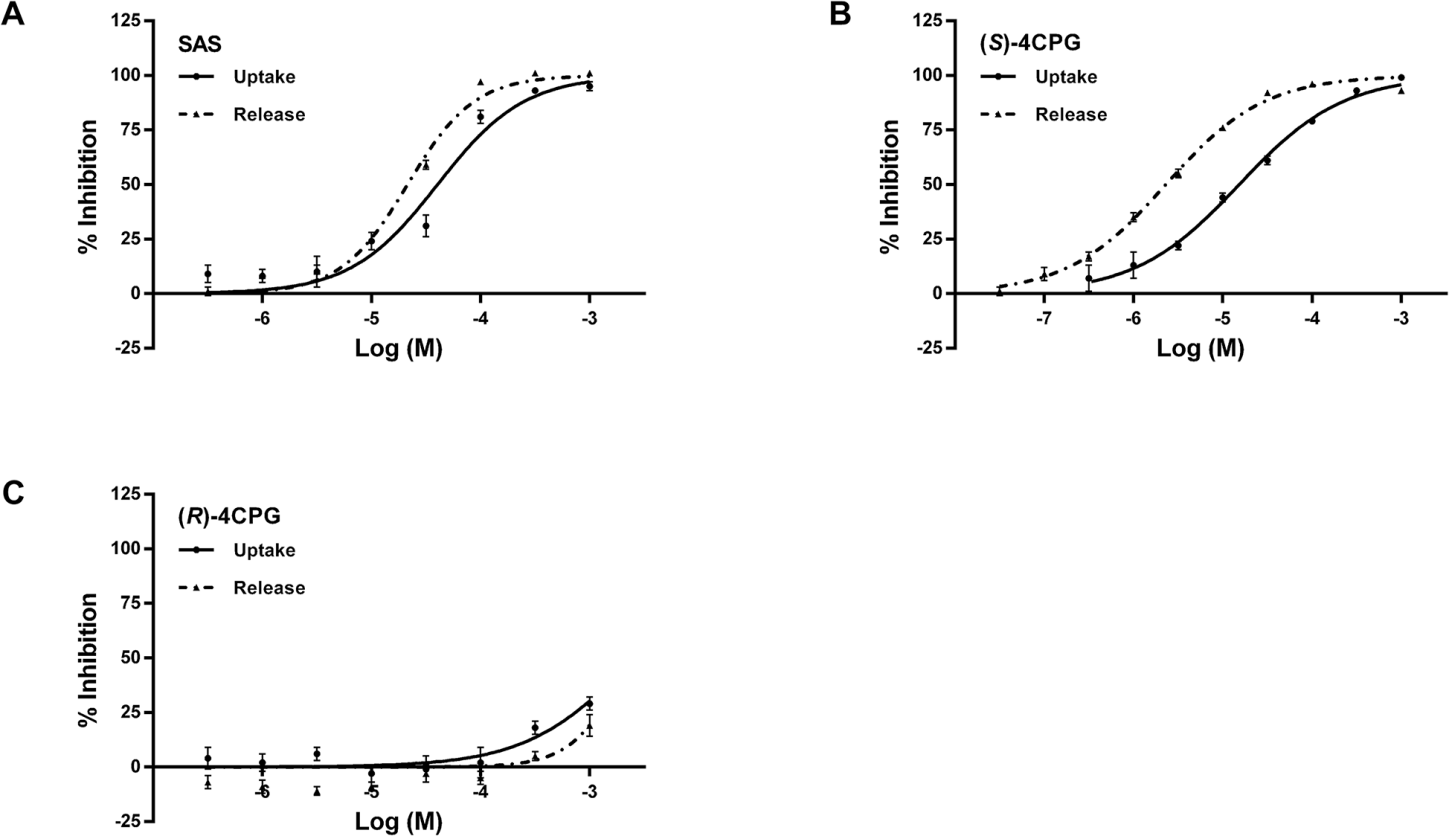
Dose responses of A. sulfasalazine (SAS), B. (*S*)-4-carboxyphenylglycine ((*S*)-4CPG) and C. (*R*)-4-carboxyphenylglycine ((*R*)-4CPG) using [^14^C]-cystine uptake and cystine-induced glutamate release assays in CCF-STTG-1 cells. Cystine uptake was conducted at 37°C for 15 min using 80μM cystine, and at a specific activity of 5.631 μCi/μmol, in the presence and absence of inhibitors. At the end of the experiment, cells were lysed and the radioactivity in the cells measured using a scintillation counter and normalized to the protein contents. Cystine-induced glutamate released over 2h at 37°C upon the addition of 80 μM cystine, in the presence and absence of inhibitors, was measured directly using glutamate oxidase, horse radish peroxidase and Amplex UltraRed and the rate of change of fluorescence monitored at ex 530, em 590. For both assays, results were normalized to totals and blanks and the IC_50_ values determined as a function of the normalized values. Data are an average of 2–6 independent experiments.

Known inhibitors of system x_c_^-^, SAS and (*S*)-4CPG, were evaluated in both assays using CCF-STTG-1 cells as the source of the antiporter. Selectivity was verified with the known inactive enantiomer, (*R*)-4CPG. While both SAS and (*S*)-4CPG inhibited cystine transport and cystine-induced glutamate release through system x_c_^-^, (*R*)-4CPG did not (Table 2, Fig 5). The IC_50_ values for inhibition of glutamate release were lower than the IC_50_ values for inhibition of cystine uptake within each cell line (Table 2). On the other hand, the IC_50_ values of (*S*)-4CPG, (*R*)-4CPG, SAS and the co-transporter, glutamate (in the uptake assay), were similar across multiple cell lines (CCF-STTG-1, U-87 MG, IMR-90, H4) regardless of the stoichiometry of cystine uptake to glutamate efflux (Tables 1 and 2). Furthermore, the IC_50_ values of SAS and (*S*)-4CPG were the same in the presence or absence of sodium (Table 2) or in a nominally, calcium-free buffer (no added calcium, formulation: S1 Table; data: S3 Table). Previously published analogs of SAS [25] were also evaluated in the release assay using CCF-STTG-1 cells as the source of system x_c_^-^. These yielded IC_50_ values comparable to the published reports, with similar rank order of potencies within the same chemical series (S4 Table).]

**Table 2:** IC_50_ values (μM) of (*S*)-, (*R*)-4CPG, glutamate and sulfasalazine (SAS) in [^14^C]-cystine uptake and cystine-induced glutamate release assays using different cell sources of the antiporter and under different buffering conditions. Cystine uptake and glutamate release assays were carried out within the linear range for both processes; 15 min and 2 h, respectively.

UB (-Na^+^)							
	**Cystine Uptake**				**Glutamate Release**		
**Lines tested**	**(*S*)-4CPG**	**(*R*)-4CPG**	**Glutamate**	**SAS**	**(*S*)-4CPG**	**(*R*)-4CPG**	**SAS**
CCF-STTG-1	10 ± 0.6	> 500	600 ± 30	40 ± 1	2 ± 0.4	> 100	20 ± 0.7
U-87 MG*	20 ± 1	> 1000	300 ± 10	30 ± 2	-	-	-
IMR-90*	30 ± 1	> 500	200 ± 9	40 ± 2	-	-	-
H4	20 ± 0.4	> 500	600 ± 20	40 ± 1	5 ± 0.7	> 100	30 ± 0.6
**EBSS (+Na** ^**+**^ **)**							
	**Cystine Uptake**				**Glutamate Release**		
**Lines tested**	**(*S*)-4CPG**	**(*R*)-4CPG**	**Glutamate**	**SAS**	**(*S*)-4CPG**	**(*R*)-4CPG**	**SAS**
CCF-STTG-1	20 ± 0.9	> 500	500 ± 30	40 ± 3	2 ± 0.05	> 100	20 ± 0.5
U-87 MG	20 ± 1	> 500	200 ± 10	30 ± 1	3 ± 0.4	> 100	20 ± 0.4
IMR-90	50 ± 2	> 500	300 ± 30	50 ± 2	5 ± 0.7	> 100	5 ± 0.4
H4	20 ± 2	> 500	500 ± 10	40 ± 1	8 ± 1.0	> 100	20 ± 0.3

* No cystine-induced glutamate release in these lines under sodium-free conditions.

### Comparison of the rate of cystine influx vs. the rate of glutamate efflux shows faster rate of cystine influx in glioma cells

**Fig 6:**
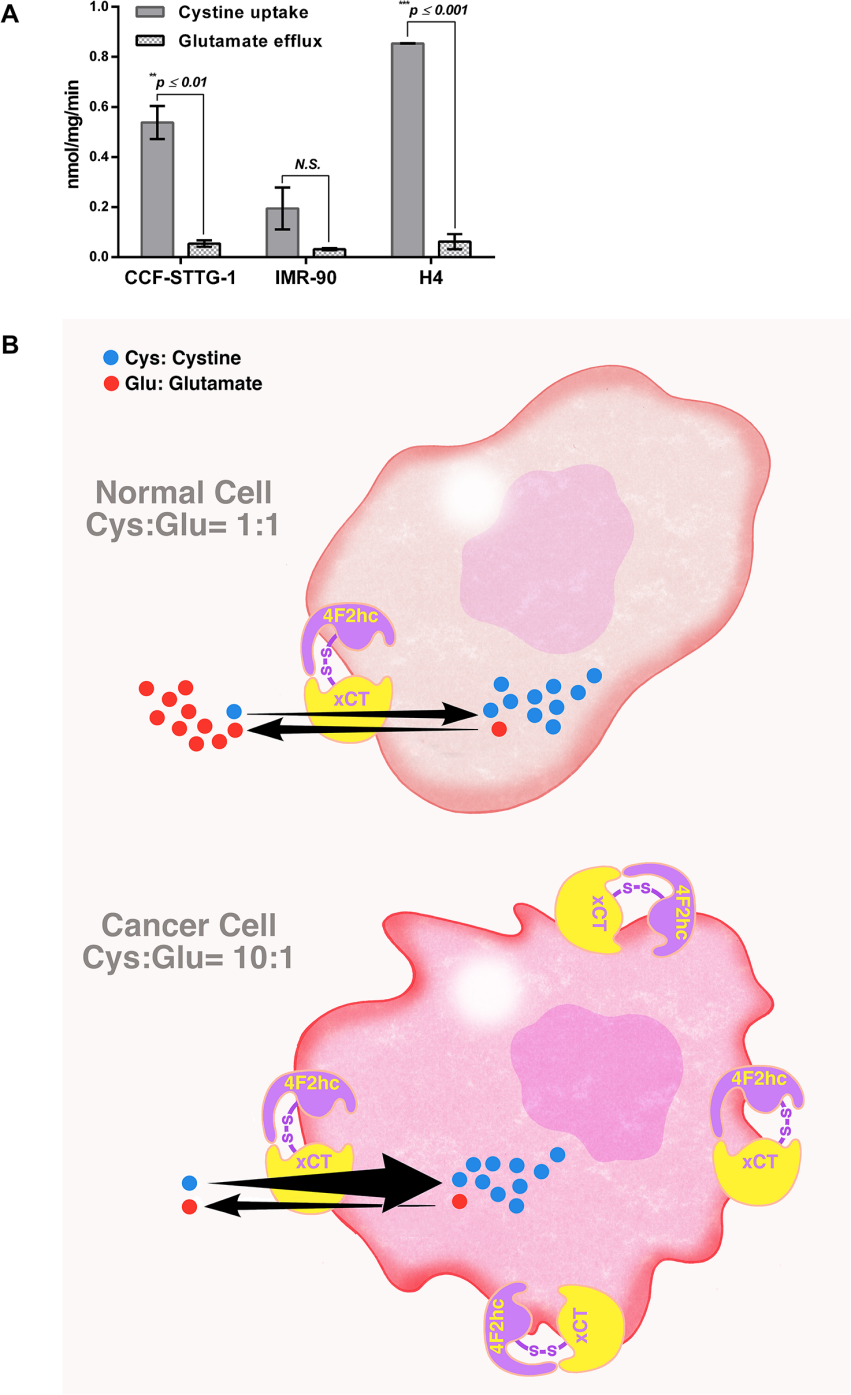
A. Rates of [^14^C]-cystine influx and cystine-induced glutamate efflux in CCF-STTG-1, IMR-90 and H4 cells. Experiments were performed using previously published protocols [29] with minor modifications. Cells were seeded at 0.04 x 10^6^ cells per 96-well, grown to confluence (Day 3), washed with pre-warmed PBS, and experiments initiated upon the addition of 80 μM cystine with or without L-[3,3’-^14^C]-cystine (5.631 μCi/μmol). Cystine uptake was terminated after 2 min and cystine-induced glutamate release measured after 30 min. The rates of cystine uptake and cystine-induced glutamate release were calculated from an average of 2 independent experiments with 16 determinations per experiment and normalized to time and protein content (nmol/mg/min ± SD); ^**^ *p < 0*.*001 vs*. *rate of glutamate efflux; N*.*S*.*—not significant*. B. Proposed schema for system xc- activity and glutamate metabolism in normal (top) and cancer cells (bottom). In both normal and cancer cells, the affinities (K_m_) of system x_c_^-^ for extracellular cystine and intracellular glutamate are 0.05 mM and 7.5 mM, respectively [29]. Normal cells exhibit a 1:1 ratio of cystine uptake to glutamate release. Cancer cells exhibit a ≥ 10:1 ratio of cystine uptake to glutamate release.

The rates of [^14^C]-cystine influx and cystine-induced glutamate efflux were closely examined using CCF-STTG-1 (grade IV astrocytoma) cells in order to understand the reasons for the lower IC_50_ values and longer time-dependence in the cystine-induced glutamate release assay. Cells were seeded similarly (40,000 cells/well) and both experiments initiated (on Day 3) upon the addition of the same concentration of cystine (80 μM) under similar experimental conditions as published originally [29]. The experiments were repeated also using IMR-90 fibroblast cells derived from normal embryonic lung and a second cancer line, the H4 neuroglioma cells. IMR-90 cells showed a trend towards faster cystine uptake versus glutamate release but the difference was not significant (Fig 6A). In contrast, both CCF-STTG-1 and H4 cancer cells showed a statistically significant, 10 to 14-fold difference between the cystine uptake and cystine-induced glutamate efflux rates.

## Discussion

System x_c_^-^ is a promising therapeutic target due to its ability to play a critical role in cancer therapy, via modulation of both glutathione biosynthesis and glutamate release, and in neurodegenerative diseases via regulation of extracellular glutamate. However, system x_c_^-^ has received little attention partly due to the potential safety risk associated with blocking glutathione biosynthesis and partly due to the lack of selective and potent small molecule system x_c_^-^ inhibitors. Indeed, while some cell-based studies indicate that loss of system x_c_^-^ function leads to cytotoxic effects [34, 35], two different strains of mice lacking system x_c_^-^ function have been reported to be healthy in appearance and fertile [36, 37]. Furthermore, siRNAs against system x_c_^-^ have not shown any major side effects [12]. Here, as a first step to identify new scaffolds for inhibitor design, we developed a high throughput screening assay against system x_c_^-^. We report on the characterization and implementation of a [^14^C]-cystine uptake assay and a cystine-induced glutamate release assay.

Various transport assays used to identify system x_c_^-^ activity have included [^3^H]-cystine [1], [^14^C]-cystine [28], [^35^S]-cystine [3], [^3^H]-glutamate uptake assays [7, 24] as well as cystine-induced glutamate release assays [7, 24, 29]. However, since [^35^S] has a relatively short half-life of 87 days [38], [^3^H]-cystine is no longer commercially available and the physiological flux via system x_c_^-^ involves the entry of L-cystine and the exit of L-glutamate [28], [^35^S]-, [^3^H]-cystine and [^3^H]-glutamate uptake assays were not considered as viable options. Instead, a direct, [^14^C]-cystine uptake assay [28] and a cystine-induced glutamate release assay [7, 24, 29] were used to define system x_c_^-^ activity.

A second key determinant in the characterization of the assay was the source of system x_c_^-^. The best cell source of the transporter was determined based on system x_c_^-^ activity measured by cystine-induced glutamate release. Multiple non-neuronal, non-adherent and neuronal, adherent human cells were assessed for system x_c_^-^ activity. The highest expression and activity levels were observed in the newly acquired human neuroglioma cells (H4) and the human astrocytoma cells (CCF-STTG-1, Table 1). There was a strong correlation between xCT/*SLC7A11* mRNA expression and transporter activity (0.96) suggesting that the control of gene expression occurred primarily at the level of transcription rather than at the level of translation.

System x_c_^-^ activity, as defined by [^14^C]-cystine uptake using CCF-STTG-1 cells, followed Michaelis-Menten kinetics, with apparent *K*_*m*_ consistent with earlier reports [1, 3, 28], and exhibited protein-, time- and temperature-dependence (Fig 1). Uptake was linear up to 15 min (time course at 37°C; Fig 1D) suggesting no immediate egress of the label from the cells. However, at longer incubations (> 15 min), [^14^C]-cystine uptake resulted in lower specific rates (data not shown), consistent with [^14^C]- efflux out of cells via cysteine formation [3, 39]. Known inhibitors of system x_c_^-^ [24, 26] gave similar IC_50_ values as those reported previously [9, 40] (Table 2, Fig 5) corroborating earlier reports of Na^+^-independent L-cystine uptake [3, 28]. The co-transporter, glutamate, also inhibited cystine uptake with comparable inhibitory potency across all cell types (IC_50_: 200–600 μM; Table 2) irrespective of sodium. Additionally, using previously published protocols [1], the IC_50_ for glutamate was also consistent with its reported K_i_ against cystine uptake (in IMR-90 cells, S5 Table). However, as a HTS assay, the uptake assay is limited in its scope by (i) the sensitivity of the transport to temperature of the wash and assay buffers (Fig 1E), (ii) the necessity for multiple washes before and after transport, (iii) the need for transfer of cell lysate to a solid scintillator plate and (iv) the cost of the label. Consequently, it was decided that [^14^C]-cystine uptake assay would be most useful in a HTS campaign as a confirmatory assay rather than as a primary screening assay.

An alternative way to assess system x_c_^-^ activity was to measure cystine-induced glutamate release [7, 24, 29]. System x_c_^-^ activity as measured by the coupling of the glutamate efflux via the transporter to its extracellular metabolism by GO [33] provided greater sensitivity and most importantly, direct, online measurement capabilities (i.e., cystine-induced glutamate release and its detection made in the same well) than previously described glutamate analysis [29] or GDH mediated methods [7, 24] (S1 Fig). Cystine-induced glutamate release through system x_c_^-^ also exhibited Michaelis-Menten behavior (Fig 2A) with apparent *K*_*m*_ similar to that obtained using the [^14^C]-cystine uptake assay and comparable to those reported previously [3, 29]. This is not surprising since, in each case, the binding affinity (*K*_*m*_) that is measured is the affinity of the transporter for cystine regardless of the different end point measurements. At 37°C, cystine-induced glutamate release also exhibited protein- and time-dependence (Fig 2). Interestingly, glutamate release was linear over a 4h period (Fig 2D), and potentially longer [3], in contrast to the time-dependence data for cystine uptake (Fig 1D). This disparity could be due to the time taken by intercellular glutamate precursors to generate and subsequently release glutamate [3]. For the 384-well assay, additional optimization in terms of wash buffer temperature, number of washes, wash volumes, aspiration and dispense speeds resulted in minimal assay variability between different sections of the plate and across the entire plate (*Z’* values of 0.5–0.7; Fig 3). Final assay characterization using known inhibitors of system x_c_^-^ [24, 26], in Na^+^-free and Na^+^-containing buffers, resulted in similar IC_50_ values (Table 2). Also, the absence of calcium did not alter the IC_50_ values of the prototypes (S2 Table). Additionally, a pilot screen of the LOPAC and the NINDS libraries demonstrated that the logistics of the assay were in place. Unfortunately, the hit compounds were not amenable to medicinal chemistry optimization. In addition to being weak inhibitors of system x_c_^-^, the compounds exhibited low solubility (clog *P* > 5), had high molecular weights (except compound TTNPB), or were expected to have poor blood brain barrier penetrability (Fig 4).

Further validation with previously published SAS analogs [25] yielded comparable values and similar rank order of potency in both the glutamate release and uptake assays (S4 Table). Of note, were the relatively lower IC_50_ values of most compounds in the glutamate release assays as compared to the uptake assays (Tables 2 and S4, Fig 5). One plausible explanation is that cystine uptake may be mediated through other routes of entry besides system x_c_^-^ [41]. That cystine uptake occurs without concomitant glutamate release in both the U-87 MG human glioblastoma cells and in the IMR-90 human fibroblast cells in the absence of sodium (Table 2) suggests a different pathway for cystine ingress. An alternative, though not mutually exclusive, explanation for the discrepancy in IC_50_ values between the two assays is that, in the glutamate release assay, a system x_c_^-^ inhibitor could be blocking both the influx of cystine and the subsequent cystine-induced efflux of glutamate, resulting in the appearance of a more potent system x_c_^-^ inhibitor (lower IC_50_ value); whereas in the uptake assay, the IC_50_ values are reflective of only the direct blockage of [^14^C]-cystine influx via the antiporter.

A closer look at the two assays for system x_c_^-^ activity measurement revealed that, in CCF-STTG-1 cells, the rate of entry of cystine was more than 10-fold higher than the rate of exodus of glutamate (Fig 6A). Careful scrutiny of an earlier work with CCF-STTG-1 cells shows saturation of cystine uptake occurs within 20 min while cystine-induced glutamate release continues to occur even at 14 h [3] suggesting a faster cystine uptake rate than the glutamate efflux rate in accordance with our results. However, these results are in contrast to the 1:1 ratio reported previously for human fibroblast cells derived from normal embryonic lung (IMR-90 cells) [29]. A repeat of the experiments under originally published conditions [29], i.e. uptake at 2 min and release at 30 min, revealed a similar trend in normal IMR-90 cells as with glioma cells (Fig 6A). However, there was no significant difference between the two rates, in agreement with Bannai’s earlier results [29]. In contrast, both the CCF-STTG-1 and H4 cancer cells showed a statistically significant, 10 to 14-fold higher cystine uptake to glutamate efflux rate. These results are consistent with the notion that glioma cells have a unique requirement for extracellular cystine for intracellular cysteine synthesis [8]. Furthermore, whereas the affinity (K_m_) of system x_c_^-^ for extracellular cystine is 0.05 mM, the affinity of the same transporter for intracellular glutamate is 7.5 mM [29]. While it is reasonable to assume that the affinities (K_m_) of extracellular cystine and intracellular glutamate for the transporter is similar across different cell types, the amount of intracellular glutamate available for cystine-induced release may also be different due to the distinct metabolic needs of normal and cancer cells (Fig 6B). Recent evidence suggests that, in cancer cells, glutamate maybe re-routed as an oxidative substrate for the Krebs (tricarboxylic acid (TCA)) cycle to produce ATP [42–45], resulting in reduced glutamate availability for release. Since the affinity of intracellular glutamate for the transporter is much lower than that of extracellular cystine for the same transporter, much less glutamate gets out resulting in lower than expected rate of glutamate efflux. The lower IC_50_ values in the glutamate release assays are consistent with also this hypothesis.

Regardless of the differences in IC_50_ values, these assays that have been characterized are designed to identify inhibitors of system x_c_^-^ that could impede cancer cell growth via disruption of GSH synthesis or prevent glutamate excitotoxicity in neurodegenerative diseases via interruption of microglial activation. Even though in these assays we follow cystine uptake and glutamate release, processes associated with system x_c_^-^, it is important to note that these assays are phenotypic and reflect many other activities of the cell. It is conceivable that compounds identified as inhibitors of system x_c_^-^ may also have an impact at many other sites, including those known to block microglial activation such as inhibitors of glutaminase, nuclear factor κB, mitogen-activated protein kinases [46] and metabotropic glutamate receptor (mGluR) ligands [47, 48]. In fact, recent evidence indicates that glutamate release through system x_c_^-^ is coupled to NADPH oxidase in microglia [49], an enzyme complex that maybe inhibited by activating mGluR’s [50].

In summary, system x_c_^-^ activity as defined by both [^14^C]-cystine uptake and cystine-induced glutamate release were successfully characterized. A close examination of the characterization data using glioma cells revealed significantly faster cystine uptake than glutamate release. The 384-well cystine-induced glutamate release assay, along with a counter screen, is effectively being implemented to carry out a HTS campaign to identify ideal system x_c_^-^ inhibitors for brain cancer and other neurodegenerative diseases associated with excess extracellular glutamate.

## Supporting Information

S1 Table Buffer formulations. (DOCX)

S2 Table Effect of 0.2% DMSO on ‘totals’ (buffer containing cystine 80 μM; units: RFU/s) from cystine-induced glutamate release experiments. (DOCX)

S3 Table Effect of calcium on specific rates and IC_50_ values of (*S*)-, (*R*)-4CPG and sulfasalazine (SAS) in cystine uptake assays. (DOCX)

S4 Table IC_50_ values of sulfasalazine (SAS) and its analogs in [^14^C]-cystine uptake and cystine-induced glutamate release assays. Compounds arranged as per rank order in [^14^C]-cystine uptake.
(DOCX)

S5 Table IC_50_ values (μM) of (*S*)-, (*R*)-4CPG, glutamate and sulfasalazine (SAS) in [^14^C]-cystine uptake (2 min) and cystine-induced glutamate release (30 min) assays using CCF-STTG-1, IMR-90 and H4 cells as per previously published methods [29]. (DOCX)

S6 Table Cystine influx and glutamate efflux rates (nmol/mg/min) in CCF-STTG-1, IMR-90 and H4 cells as per previously published methods [29]. (DOCX)

S1 Fig Cystine-induced glutamate detection in glioma cells using either A. glutamate dehydrogenase (5 U/mL) and NADP+ (500 μM) or B. glutamate oxidase (0.04 U/mL), HRP (0.125 U/mL) and Amplex UltraRed (50 μM). In both instances, assays were conducted in Tris buffer (100 mM, pH 7.4) and the rate of change of fluorescence monitored: NADPH formation at ex 340, em 460 and resorufin formation at ex 530, em 590. *Z*’ values, specific rates and IC_50_ values of (*S*)-4CPG and (*R*)-4CPG are shown in the insets.
(TIF)

S1 File Data Table 1. (XLSX)

S2 File Data Table 2. (XLSX)

S3 File Data Fig 1A and 1B. (XLSX)

S4 File Data Fig 1C. (XLSX)

S5 File Data Fig 1D. (XLSX)

S6 File Data Fig 1E. (XLSX)

S7 File Data Fig 2A and 2B. (XLSX)

S8 File Data Fig 2C. (XLSX)

S9 File Data Fig 2D. (XLSX)

S10 File Data Fig 3. (XLSX)

S11 File Data Fig 4. (XLSX)

S12 File Data Fig 5A. (PZF)

S13 File Data Fig 5B. (PZF)

S14 File Data Fig 5C. (PZF)

S15 File Data Fig 6A, S5 Table, S6 Table–Cystine uptake. (XLSX)

S16 File Data Fig 6A, S5 Table, S6 Table–Glutamate efflux. (XLSX)

S17 File Data S2 Table. (XLSX)

S18 File Data S3 Table. (XLSX)

S19 File Data S4 Table. (XLSX)

S20 File Data S5 Table. (XLSX)

S21 File Data S1 Fig. (XLSX)
