# Supplementary material for: High-Throughput Assay Development for Cystine-Glutamate Antiporter (xc -) Highlights Faster Cystine Uptake than Glutamate Release in Glioma Cells
Source: PLoS One. 2015 Aug 7;10(8):e0127785. doi: 10.1371/journal.pone.0127785 (PMC4529246; doi:10.1371/journal.pone.0127785)
Supplement: S1 Table — (DOCX) [file pone.0127785.s001.docx]

**S1 Table. Buffer formulations**

| **Cl^-^- dependent (Na^+^-independent) Uptake Buffer (UB):** | | | | | | | **Ca^2+^-free Uptake Buffer:** | | | | | |  |  |  |
| --- | --- | --- | --- | --- | --- | --- | --- | --- | --- | --- | --- | --- | --- | --- | --- |
|  |  | mM |  | Total ions | mM |  |  |  | | mM |  | Total ions | | | mM |
| Choline Chloride | | 137.5 |  | Na^+^ | 0.000 |  | Choline Chloride | | | 137.5 |  | Na^+^ | | | 0.000 |
| KCl |  | 5.36 |  | K^+^ | 6.130 |  | KCl |  | | 5.36 |  | K^+^ | | | 6.130 |
| KH_2_PO_4_ |  | 0.77 |  | Mg^2+^ | 0.710 |  | KH2PO4 |  | | 0.77 |  | Mg^2+^ | | | 0.710 |
| MgSO_4_.7H_2_O | | 0.71 |  | Ca^2+^ | 1.100 |  | MgSO4 7H2O | | | 0.71 |  | Ca^2+^ | | | 0.000 |
| CaCl_2_.2H_2_0 |  | 1.1 |  | Cl^-^ | 143.960 |  | CaCl2 |  | | 0 |  | Cl^-^ | | | 142.860 |
| Glucose |  | 10 |  | PO_4_^3-^ | 0.770 |  | Glucose |  | | 10 |  | PO_4_^3-^ | | | 0.770 |
| HEPES |  | 10 |  | SO_4_^2-^ | 0.710 |  | HEPES |  | | 10 |  | SO_4_^2-^ | | | 0.710 |
| **pH to 7.4 with KOH** | | |  |  |  |  | **pH to 7.4 with KOH** | | | |  |  | | |  |
|  |  |  |  |  |  |  |  |  |  | |  |  | | |  |
|  |  |  |  |  |  |  |  |  |  | |  |  | | |  |
| **Earle's Balanced Salt Solution (EBSS, Sigma E3024):** | | | | | | | |  |  | |  |  | | |  |
|  |  | mM |  | Total ions | mM |  |  |  |  | |  |  | | |  |
| NaCl |  | 116.4 |  | Na^+^ | 143.615 |  |  |  |  | |  |  | | |  |
| NaHCO_3_ |  | 26.20 |  | K^+^ | 5.360 |  |  |  |  | |  |  | | |  |
| NaH_2_PO_4_ (anhyd) | | 1.02 |  | Mg^2+^ | 0.810 |  |  |  |  | |  |  | | |  |
| KCl |  | 5.36 |  | Ca^2+^ | 1.800 |  |  |  |  | |  |  | | |  |
| MgSO_4_ (anhyd) | | 0.81 |  | Cl^-^ | 123.560 |  |  |  |  | |  |  | | |  |
| CaCl_2_.2H_2_0 |  | 1.8 |  | PO_4_^3-^ | 1.015 |  |  |  |  | |  |  | | |  |
| Glucose |  | 5.56 |  | SO_4_^2-^ | 0.810 |  |  |  |  | |  |  | | |  |
| **pH to 7.4 with NaOH** | | |  |  |  |  |  |  |  | |  |  | | |  |
|  |  |  |  |  |  |  |  |  |  | |  |  | | |  |
|  |  |  |  |  |  |  |  |  |  | |  |  | | |  |
| **Phosphate-buffered Saline (PBS):** | | | | |  |  |  |  |  | |  |  | | |  |
|  |  | mM |  | Total ions | mM |  |  |  |  | |  |  | | |  |
| NaCl |  | 137 |  | Na^+^ | 147.000 |  |  |  |  | |  |  | | |  |
| Na_2_HPO_4_ |  | 10.00 |  | K^+^ | 3.000 |  |  |  |  | |  |  | | |  |
| KCl |  | 3 |  | Mg^2+^ | 0.492 |  |  |  |  | |  |  | | |  |
| MgCl_2_.6H_2_O |  | 0.492 |  | Ca^2+^ | 0.904 |  |  |  |  | |  |  | | |  |
| CaCl_2_.2H_2_0 |  | 0.904 |  | Cl^-^ | 141.396 |  |  |  |  | |  |  | | |  |
| Glucose |  | 5.56 |  | PO_4_^3-^ | 10.000 |  |  |  |  | |  |  | | |  |
|  |  |  |  | SO_4_^2-^ | 0.000 |  |  |  |  | |  |  | | |  |
| **pH to 7.4 with HCl** | |  |  |  |  |  |  |  |  | |  |  | | |  |
