# Supplementary material for: High-Throughput Assay Development for Cystine-Glutamate Antiporter (xc -) Highlights Faster Cystine Uptake than Glutamate Release in Glioma Cells
Source: PLoS One. 2015 Aug 7;10(8):e0127785. doi: 10.1371/journal.pone.0127785 (PMC4529246; doi:10.1371/journal.pone.0127785)
Supplement: S2 Table — (DOCX) [file pone.0127785.s002.docx]

**S2 Table. Effect of 0.2% DMSO on ‘totals’ (buffer containing cystine 80 µM; units: RFU/s) from cystine-induced glutamate release experiments**

| **EBSS (+Na^+^)** | **Glutamate Release - 2 h** | | |  |
| --- | --- | --- | --- | --- |
|  | **With 0.2% DMSO** | | **Without 0.2% DMSO** | |
| **Totals (RFU/s)** | 0.685 ± 0.010 | | 0.663 ± 0.009 |  |
| **N^*^** | 2 |  | 2 |  |
|  |  |  |  |  |
| **Ttests *vs.*** |  |  |  |  |
| Totals without 0.2% DMSO | *0.10* |  |  |  |
| ^*^Each N is the average of 16 determinations | |  |  |  |
