# Supplementary material for: High-Throughput Assay Development for Cystine-Glutamate Antiporter (xc -) Highlights Faster Cystine Uptake than Glutamate Release in Glioma Cells
Source: PLoS One. 2015 Aug 7;10(8):e0127785. doi: 10.1371/journal.pone.0127785 (PMC4529246; doi:10.1371/journal.pone.0127785)
Supplement: S3 Table — (DOCX) [file pone.0127785.s003.docx]

**S3 Table. Effect of calcium on specific rates and IC_50_ values of (*S*)-, (*R*)-4CPG and sulfasalazine (SAS) in cystine uptake assays**

| **UB (-Na^+^)** | **Cystine Uptake - 15 min** | | |  |
| --- | --- | --- | --- | --- |
|  | **With Calcium** | | **Without Calcium** | |
| **Specifc rate (CPM)** | 914 ± 40 |  | 821 ± 25 |  |
| **N^*^** | 2 |  | 2 |  |
| **IC_50_ values (µM)** |  |  |  |  |
| **(*S*)-4CPG** | 15 ± 1.1 |  | 20 ± 1.6 |  |
| **(*R*)-4CPG** | > 500 |  | > 500 |  |
| **SAS** | 40 ± 1.3 |  | 40 ± 1.2 |  |
| **Ttests *vs.*** |  |  |  |  |
| Ca^2+^-free uptake | *< 0.001* |  |  |  |
| ^*^Each N is the average of 16 determinations | | |  |  |
