# Supplementary material for: High-Throughput Assay Development for Cystine-Glutamate Antiporter (xc -) Highlights Faster Cystine Uptake than Glutamate Release in Glioma Cells
Source: PLoS One. 2015 Aug 7;10(8):e0127785. doi: 10.1371/journal.pone.0127785 (PMC4529246; doi:10.1371/journal.pone.0127785)
Supplement: S4 Table — Compounds arranged as per rank order in [14C]-cystine uptake. (DOCX) [file pone.0127785.s004.docx]

**S4 Table. IC_50_ values of sulfasalazine (SAS) and its analogs in [^14^C]-cystine uptake and cystine-induced glutamate release assays. Compounds arranged as per rank order in [^14^C]-cystine uptake.**

| **Compound ID** | **Molecular Structure** | **Average IC_50_ (μM)** | |
| --- | --- | --- | --- |
|  |  | ***Uptake Assay**** | ***Release Assay*** |
| **JHU-33** | **** | 29 ± 1 | 8 ± 1 |
| **SAS** | **** | 34 ± 7 | 17 ± 1 |
| **JHU-719** |  | 65 ± 19 | 11 ± 2 |
| **JHU-298** |  | 72 ± 8 | 17 ± 3 |
| **JHU-184** |  | 195 ± 5 | 100 ± 2 |
| **JHU-822** |  | 270 | > 1000 |

*Data from Shukla K, Thomas AG, Ferraris DV, Hin N, Sattler R, et al. (2011) Inhibition of xc(-) transporter-mediated cystine uptake by sulfasalazine analogs. Bioorg Med Chem Lett 21: 6184-6187.
