# Supplementary material for: High-Throughput Assay Development for Cystine-Glutamate Antiporter (xc -) Highlights Faster Cystine Uptake than Glutamate Release in Glioma Cells
Source: PLoS One. 2015 Aug 7;10(8):e0127785. doi: 10.1371/journal.pone.0127785 (PMC4529246; doi:10.1371/journal.pone.0127785)
Supplement: S5 Table — (DOCX) [file pone.0127785.s005.docx]

**S5 Table. IC_50_ values (µM) of (*S*)-, (*R*)-4CPG, glutamate and sulfasalazine (SAS) in [^14^C]-cystine uptake (2 min) and cystine-induced glutamate release (30 min) assays using CCF-STTG-1, IMR-90 and H4 cells as per previously published methods [**[**29**](#_ENREF_29)**].**

| **PBS (+Na^+^)** |  |  |  |  |  |  |  |  |
| --- | --- | --- | --- | --- | --- | --- | --- | --- |
|  | **Cystine Uptake** | | | | **Glutamate Release** | | | |
| **Lines tested** | **(*S*)-4CPG** | **(*R*)-4CPG** | **Glutamate** | **SAS** | **(*S*)-4CPG** | **(*R*)-4CPG** | **SAS** | **Comments** |
| STTG1 | 20 ± 2 | > 500 | 100 ± 10 | 30 ± 0.6 | 3 ± 0.1 | > 100 | 20 ± 2 |  |
| IMR90 | 10 ± 0.08 | > 500 | 200 ± 3 | 20 ± 2 | 2 ± 0.4 | > 100 | 3 ± 0.2 |  |
| H4 | 20 ± 0.4 | > 500 | 200 ± 20 | 30 ± 0.8 | 10 ± 2 | > 100 | 30 ± 0.3 |  |
