# Supplementary material for: High-Throughput Assay Development for Cystine-Glutamate Antiporter (xc -) Highlights Faster Cystine Uptake than Glutamate Release in Glioma Cells
Source: PLoS One. 2015 Aug 7;10(8):e0127785. doi: 10.1371/journal.pone.0127785 (PMC4529246; doi:10.1371/journal.pone.0127785)
Supplement: S6 Table — (DOCX) [file pone.0127785.s006.docx]

**S6 Table: Cystine influx and glutamate efflux rates (nmol/mg/min) in CCF-STTG-1, IMR-90 and H4 cells as per previously published methods [**[**29**](#_ENREF_29)**].**

| **PBS (+Na^+^)** | **Cystine Uptake - 2 min** | |  | **Glutamate Release - 30 min** | |  |
| --- | --- | --- | --- | --- | --- | --- |
|  | **CCF-STTG-1** | **IMR-90** | **H4** | **CCF-STTG-1** | **IMR-90** | **H4** |
| **Average ± SD** | 0.538 ± 0.066 | 0.195 ± 0.084 | 0.854 ± 0.001 | 0.054 ± 0.013 | 0.031 ± 0.004 | 0.062 ± 0.030 |
| **N*** | 2 | 2 | 2 | 2 | 2 | 2 |
| **Ttests *vs.*** |  |  |  |  |  |  |
| Uptake |  |  |  | *0.010* | *0.110* | *0.001* |

*Each N is the average of 16 determinations
