# Supplementary figures and images for: High-Throughput Assay Development for Cystine-Glutamate Antiporter (xc -) Highlights Faster Cystine Uptake than Glutamate Release in Glioma Cells
Source: PLoS One. 2015 Aug 7;10(8):e0127785. doi: 10.1371/journal.pone.0127785 (PMC4529246; doi:10.1371/journal.pone.0127785)

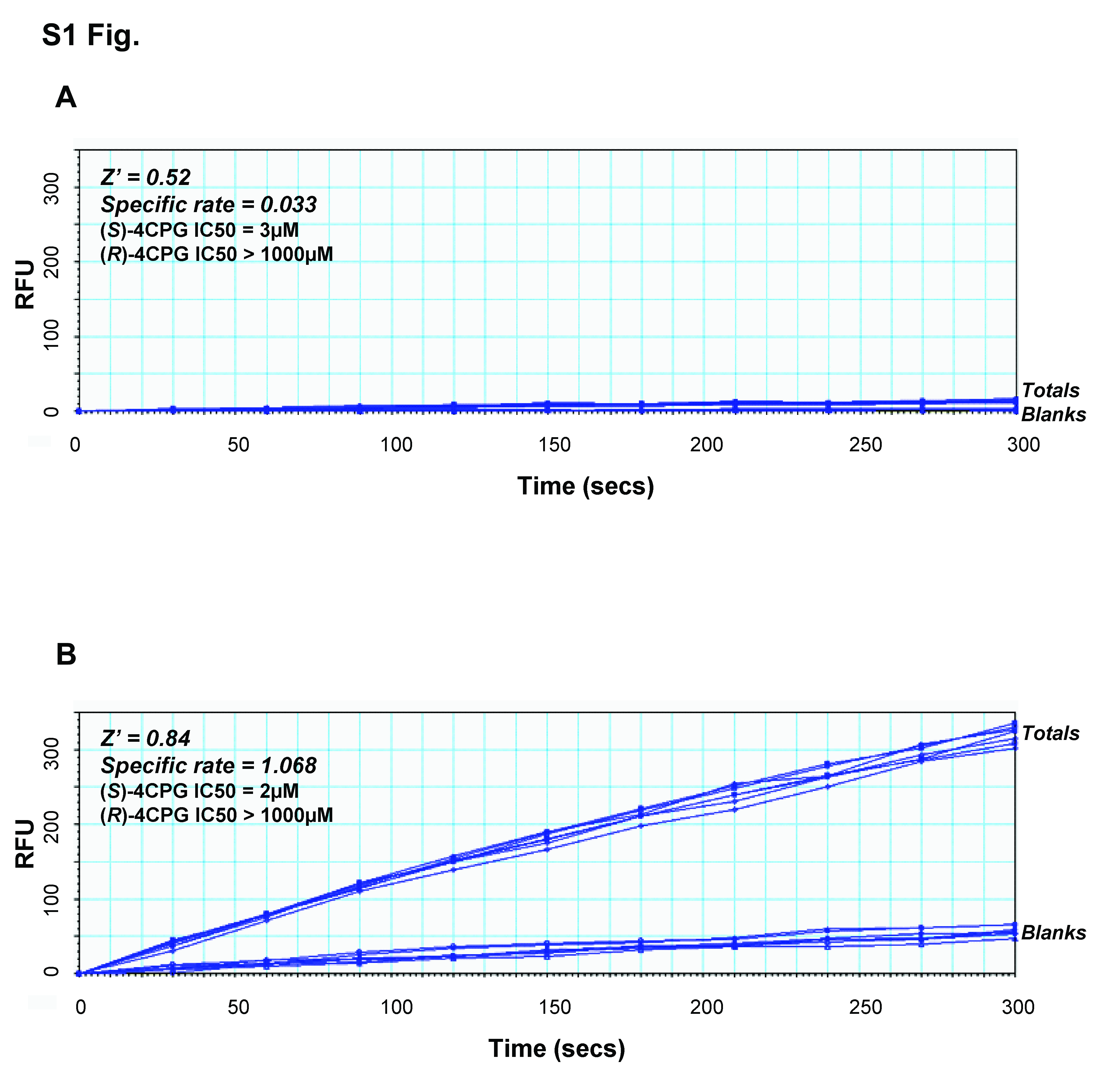

Supplement: S1 Fig — In both instances, assays were conducted in Tris buffer (100 mM, pH 7.4) and the rate of change of fluorescence monitored: NADPH formation at ex 340, em 460 and resorufin formation at ex 530, em 590. Z’ values, specific rates and IC50 values of (S)-4CPG and (R)-4CPG are shown in the insets. (TIF) [file pone.0127785.s007.tif]
